# Tetra­aqua­bis{5-[2-(1*H*-tetrazol-5-yl)ethenyl]pyrazolato-κ*N* ^2^}manganese(II) dihydrate

**DOI:** 10.1107/S1600536808012464

**Published:** 2008-05-03

**Authors:** Tuoping Hu

**Affiliations:** a Department of Chemistry, North University of China, Taiyuan, Shanxi 030051, People’s Republic of China

## Abstract

The title compound, [Mn(C_4_H_3_N_8_)_2_(H_2_O)_4_]·2H_2_O, represents the first structurally characterized transition metal complex of the 1,2-bis­(tetra­zol-5-yl)ethene ligand. The complex mol­ecule occupies a special position on an inversion centre and the Mn atom has a tetra­gonally distorted octa­hedral coordination. The bis­(tetra­zolyl)ethene ligand is planar within 0.0366 (7) Å. All ‘active’ H atoms participate in hydrogen bonds, which link mol­ecules of the complex and the uncoordinated water mol­ecules into an infinite three-dimensional framework.

## Related literature

For related literature, see: Huang *et al.* (2005 ▶); Demko & Sharpless (2001 ▶).
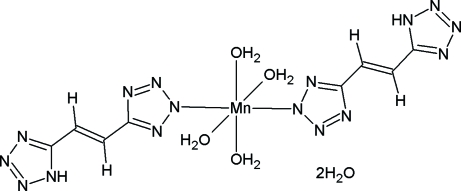


## Experimental

### 

#### Crystal data

[Mn(C_4_H_3_N_8_)_2_(H_2_O)_4_]·2H_2_O
*M* *_r_* = 489.32
Triclinic, 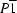

*a* = 6.2296 (2) Å
*b* = 7.0093 (2) Å
*c* = 12.1212 (3) Å
α = 84.405 (1)°
β = 89.457 (1)°
γ = 67.016 (1)°
*V* = 484.70 (2) Å^3^
*Z* = 1
Mo *K*α radiation
μ = 0.75 mm^−1^
*T* = 273 (2) K
0.36 × 0.28 × 0.16 mm

#### Data collection

Bruker SMART CCD area-detector diffractometer
Absorption correction: multi-scan (*SADABS*; Sheldrick, 1996 ▶) *T*_min_ = 0.717, *T*_max_ = 0.887
9107 measured reflections
3246 independent reflections
3149 reflections with *I* > 2σ(*I*)
*R*_int_ = 0.015

#### Refinement

*R*[*F* ^2^ > 2σ(*F* ^2^)] = 0.022
*wR*(*F* ^2^) = 0.068
*S* = 1.04
3246 reflections
179 parameters
All H-atom parameters refined
Δρ_max_ = 0.37 e Å^−3^
Δρ_min_ = −0.22 e Å^−3^

### 

Data collection: *SMART* (Bruker, 2007 ▶); cell refinement: *SAINT-Plus* (Bruker, 2007 ▶); data reduction: *SAINT-Plus*; program(s) used to solve structure: *SHELXS97* (Sheldrick, 2008 ▶); program(s) used to refine structure: *SHELXL97* (Sheldrick, 2008 ▶); molecular graphics: *SHELXTL* (Sheldrick, 2008 ▶); software used to prepare material for publication: *SHELXTL*.

## Supplementary Material

Crystal structure: contains datablocks global, I. DOI: 10.1107/S1600536808012464/ya2068sup1.cif

Structure factors: contains datablocks I. DOI: 10.1107/S1600536808012464/ya2068Isup2.hkl

Additional supplementary materials:  crystallographic information; 3D view; checkCIF report

## Figures and Tables

**Table:** 

Mn1—O1	2.1923 (7)
Mn1—O2	2.1835 (8)
Mn1—N2	2.2538 (7)

**Table:** 

O2—Mn1—O1	84.57 (3)
O2—Mn1—N2	91.07 (3)
O1—Mn1—N2	90.24 (3)

**Table 2:** Hydrogen-bond geometry (Å, °)

*D*—H⋯*A*	*D*—H	H⋯*A*	*D*⋯*A*	*D*—H⋯*A*
O1—H1*B*⋯N1^i^	0.833 (18)	2.021 (18)	2.8419 (10)	168.8 (17)
O1—H1*A*⋯O3^ii^	0.823 (18)	1.940 (18)	2.7599 (11)	174.0 (16)
O2—H2*B*⋯O3^iii^	0.790 (18)	1.996 (19)	2.7797 (11)	171.4 (18)
O2—H2*A*⋯N6^iv^	0.81 (2)	2.04 (2)	2.8472 (10)	173.7 (18)
O3—H3*A*⋯N5^v^	0.82 (2)	2.09 (2)	2.8922 (11)	164.2 (19)
O3—H3*B*⋯O1	0.80 (2)	2.30 (2)	3.0693 (12)	160 (2)
N8—H8⋯N4^vi^	0.926 (18)	1.792 (18)	2.7171 (10)	176.6 (16)

Symmetry codes: (i) 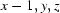; (ii) 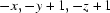; (iii) 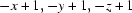; (iv) 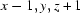; (v) 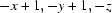; (vi) 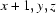.

**Fig. 1.**
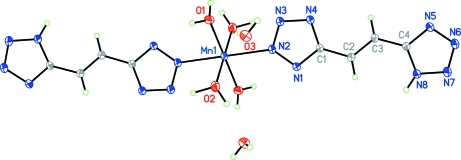
Molecular structure showing 50% probability displacement ellipsoids. The unlabeled atoms are derived from the reference atoms by means of the (1 - x, -y, 1 - z) symmetry transformation..

**Fig. 2.**
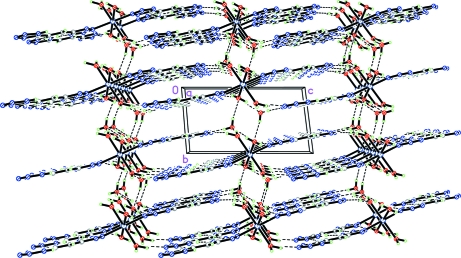
Packing diagram viewed down the a axis, The hydrogen bonds are shown as dotted lines.

## supplementary crystallographic information

### Comment

The 1,2-bis(1,2,3,4-tetrazol-5-yl)ethene ligand (H_2_BTAE) was previously reported in its twice deprotonated form in the cystal structure of its sodium salt pentahydrate (Huang *et al.*, 2005). The present paper provides the fist example of its structurally characterized complex with a transition metal; the ligand in this complex is monodeprotonated.

The molecule of Mn(H-BTAE)_2_(H_2_O)_4_ occupies a special position in the inversion centre (Fig. 1); the Mn1 atom has a tetragonally distorted octahedral coordination (Table 1). The H-BTAE ligand has essentailly planar conformation, the maximum deviation of the N6 atom from its mean plane being 0.0366 (7) Å. The geometry of the ligand is similar to the one observed in Huang *et al.* (2005).

All "active" hydrogen atoms in the structure participate in the H-bonding (Table 2); the extensive H-bond system links molecules of the complex and non-coordinated water molecules into three-dimensional infinite network (Fig. 2).

### Experimental

MnCl_2_.4H_2_O (0.5 mmol, 99 mg) and 1,2-bis(1,2,3,4-tetrazol-5-yl)ethene (1 mmol, 192 mg) (Demko & Sharpless, 2001) were added to 30 ml of water:MeOH (1:1) mixture. After stirring for 30 min at room temperature, the pH value was adjusted to 7 by 1*M* NaOH, and clear solution was allowed to evaporate in the air. Nice prism-shaped crystals of the title compound were obtained after 3 days. The crystals were filtered, washed by EtOH and dried in the air.

### Refinement

All H atoms were located in the difference map and refined isotropically [O—H 0.79 (2)–0.83 (2) Å; C—H 0.91 (1) and 0.96 (1) Å; N—H 0.93 (2) Å].

### Crystal data

**Table:**

[Mn(C_4_H_3_N_8_)_2_(H_2_O)_4_]·2H_2_O	*Z* = 1
*M**_r_* = 489.32	*F*_000_ = 251
Triclinic, *P*1	*D*_x_ = 1.676 Mg m^−^^3^
Hall symbol: -P 1	Mo *K*α radiation λ = 0.71073 Å
*a* = 6.2296 (2) Å	Cell parameters from 7983 reflections
*b* = 7.0093 (2) Å	θ = 3.2–33.5º
*c* = 12.1212 (3) Å	µ = 0.75 mm^−^^1^
α = 84.405 (1)º	*T* = 273 (2) K
β = 89.457 (1)º	Prism, brown
γ = 67.016 (1)º	0.36 × 0.28 × 0.16 mm
*V* = 484.70 (2) Å^3^	

### Data collection

**Table:**

Bruker SMART CCD area-detector diffractometer	3246 independent reflections
Radiation source: fine-focus sealed tube	3149 reflections with *I* > 2σ(*I*)
Monochromator: graphite	*R*_int_ = 0.015
*T* = 273(2) K	θ_max_ = 33.5º
φ and ω scans	θ_min_ = 3.2º
Absorption correction: multi-scan(SADABS; Sheldrick, 1996)	*h* = −9→9
*T*_min_ = 0.717, *T*_max_ = 0.887	*k* = −10→10
9107 measured reflections	*l* = −16→18

### Refinement

**Table:**

Refinement on *F*^2^	Hydrogen site location: difference Fourier map
Least-squares matrix: full	All H-atom parameters refined
*R*[*F*^2^ > 2σ(*F*^2^)] = 0.022	*w* = 1/[σ^2^(*F*_o_^2^) + (0.039*P*)^2^ + 0.093*P*] where *P* = (*F*_o_^2^ + 2*F*_c_^2^)/3
*wR*(*F*^2^) = 0.068	(Δ/σ)_max_ < 0.001
*S* = 1.05	Δρ_max_ = 0.37 e Å^−^^3^
3246 reflections	Δρ_min_ = −0.22 e Å^−^^3^
179 parameters	Extinction correction: SHELXL97 (Sheldrick, 2008), Fc^*^=kFc[1+0.001xFc^2^λ^3^/sin(2θ)]^-1/4^
Primary atom site location: structure-invariant direct methods	Extinction coefficient: 0.022 (3)
Secondary atom site location: difference Fourier map	

### Special details

**Table:**

Experimental. H atoms were located on intermediate difference Fourier map
Geometry. All e.s.d.'s (except the e.s.d. in the dihedral angle between two l.s. planes) are estimated using the full covariance matrix. The cell e.s.d.'s are taken into account individually in the estimation of e.s.d.'s in distances, angles and torsion angles; correlations between e.s.d.'s in cell parameters are only used when they are defined by crystal symmetry. An approximate (isotropic) treatment of cell e.s.d.'s is used for estimating e.s.d.'s involving l.s. planes.
Refinement. Refinement of *F*^2^ against ALL reflections. The weighted *R*-factor *wR* and goodness of fit *S* are based on *F*^2^, conventional *R*-factors *R* are based on *F*, with *F* set to zero for negative *F*^2^. The threshold expression of *F*^2^ > σ(*F*^2^) is used only for calculating *R*-factors(gt) *etc*. and is not relevant to the choice of reflections for refinement. *R*-factors based on *F*^2^ are statistically about twice as large as those based on *F*, and *R*- factors based on ALL data will be even larger.

### Fractional atomic coordinates and isotropic or equivalent isotropic displacement parameters (Å^2^)

**Table:**

	*x*	*y*	*z*	*U*_iso_*/*U*_eq_	
Mn1	0.5000	0.0000	0.5000	0.02323 (6)	
O1	0.13139 (12)	0.18659 (13)	0.45633 (6)	0.03342 (15)	
O2	0.48182 (15)	0.26842 (14)	0.58308 (6)	0.03732 (17)	
O3	0.15295 (14)	0.60925 (13)	0.38023 (6)	0.03452 (15)	
H3A	0.108 (4)	0.639 (3)	0.3151 (17)	0.063 (5)*	
H3B	0.180 (4)	0.488 (3)	0.3935 (17)	0.068 (6)*	
N1	0.80858 (13)	0.13919 (13)	0.30839 (6)	0.02546 (14)	
N2	0.60467 (13)	0.13162 (13)	0.34123 (6)	0.02647 (14)	
N3	0.46087 (14)	0.17201 (14)	0.25572 (6)	0.02953 (16)	
N4	0.56698 (13)	0.20725 (13)	0.16459 (6)	0.02663 (15)	
N5	1.08450 (14)	0.30165 (13)	−0.16586 (6)	0.02657 (14)	
N6	1.28887 (14)	0.30586 (14)	−0.20234 (6)	0.02983 (16)	
N7	1.43373 (14)	0.27581 (14)	−0.12043 (6)	0.03067 (16)	
N8	1.32424 (13)	0.25048 (12)	−0.02771 (6)	0.02534 (14)	
C1	0.78023 (14)	0.18638 (13)	0.19858 (6)	0.02208 (14)	
C2	0.96111 (15)	0.20610 (14)	0.12755 (7)	0.02469 (15)	
C3	0.93208 (15)	0.24900 (14)	0.01755 (7)	0.02401 (15)	
C4	1.11010 (14)	0.26607 (13)	−0.05597 (6)	0.02185 (14)	
H3	0.794 (2)	0.271 (2)	−0.0175 (12)	0.035 (3)*	
H2	1.104 (3)	0.186 (2)	0.1658 (12)	0.039 (4)*	
H8	1.405 (3)	0.232 (3)	0.0389 (15)	0.057 (5)*	
H1A	0.047 (3)	0.239 (3)	0.5070 (15)	0.046 (4)*	
H1B	0.050 (3)	0.155 (3)	0.4134 (15)	0.055 (5)*	
H2B	0.587 (3)	0.304 (3)	0.5865 (15)	0.052 (4)*	
H2A	0.417 (3)	0.280 (3)	0.6422 (17)	0.057 (5)*	

### Atomic displacement parameters (Å^2^)

**Table:**

	*U*^11^	*U*^22^	*U*^33^	*U*^12^	*U*^13^	*U*^23^
Mn1	0.02135 (10)	0.03467 (10)	0.01477 (8)	−0.01276 (7)	0.00178 (6)	−0.00004 (6)
O1	0.0225 (3)	0.0535 (4)	0.0236 (3)	−0.0133 (3)	−0.0009 (2)	−0.0078 (3)
O2	0.0443 (4)	0.0551 (5)	0.0264 (3)	−0.0324 (4)	0.0122 (3)	−0.0141 (3)
O3	0.0370 (4)	0.0392 (4)	0.0279 (3)	−0.0154 (3)	−0.0016 (3)	−0.0036 (3)
N1	0.0241 (3)	0.0396 (4)	0.0164 (3)	−0.0169 (3)	0.0007 (2)	−0.0007 (2)
N2	0.0249 (3)	0.0414 (4)	0.0161 (3)	−0.0170 (3)	0.0019 (2)	0.0003 (3)
N3	0.0255 (3)	0.0484 (4)	0.0179 (3)	−0.0190 (3)	0.0011 (2)	0.0012 (3)
N4	0.0250 (3)	0.0419 (4)	0.0161 (3)	−0.0174 (3)	0.0002 (2)	0.0011 (3)
N5	0.0283 (3)	0.0380 (4)	0.0167 (3)	−0.0167 (3)	0.0015 (2)	−0.0020 (3)
N6	0.0302 (4)	0.0437 (4)	0.0185 (3)	−0.0178 (3)	0.0051 (3)	−0.0027 (3)
N7	0.0268 (4)	0.0464 (4)	0.0213 (3)	−0.0174 (3)	0.0052 (3)	−0.0024 (3)
N8	0.0230 (3)	0.0373 (4)	0.0172 (3)	−0.0138 (3)	0.0015 (2)	−0.0006 (3)
C1	0.0232 (3)	0.0296 (3)	0.0159 (3)	−0.0133 (3)	0.0014 (2)	−0.0012 (2)
C2	0.0236 (4)	0.0351 (4)	0.0190 (3)	−0.0158 (3)	0.0024 (3)	−0.0014 (3)
C3	0.0234 (4)	0.0332 (4)	0.0189 (3)	−0.0150 (3)	0.0025 (3)	−0.0019 (3)
C4	0.0234 (3)	0.0277 (3)	0.0164 (3)	−0.0122 (3)	0.0017 (2)	−0.0021 (2)

### Geometric parameters (Å, °)

**Table:**

Mn1—O2^i^	2.1835 (8)	N2—N3	1.3112 (10)
Mn1—O1	2.1923 (7)	N3—N4	1.3336 (9)
Mn1—O2	2.1835 (8)	N4—C1	1.3427 (11)
Mn1—O1^i^	2.1923 (7)	N5—C4	1.3304 (10)
Mn1—N2^i^	2.2538 (7)	N5—N6	1.3541 (10)
Mn1—N2	2.2538 (7)	N6—N7	1.2931 (11)
O1—H1A	0.823 (18)	N7—N8	1.3420 (10)
O1—H1B	0.833 (18)	N8—C4	1.3403 (11)
O2—H2B	0.790 (18)	N8—H8	0.926 (18)
O2—H2A	0.81 (2)	C1—C2	1.4526 (11)
O3—H3A	0.82 (2)	C2—C3	1.3360 (11)
O3—H3B	0.80 (2)	C2—H2	0.963 (15)
N1—C1	1.3361 (10)	C3—C4	1.4499 (11)
N1—N2	1.3468 (10)	C3—H3	0.914 (14)
			
O2^i^—Mn1—O2	180.0	N3—N2—N1	110.34 (6)
O2^i^—Mn1—O1	95.43 (3)	N3—N2—Mn1	115.77 (5)
O2—Mn1—O1	84.57 (3)	N1—N2—Mn1	132.31 (6)
O2^i^—Mn1—O1^i^	84.57 (3)	N2—N3—N4	108.57 (7)
O2—Mn1—O1^i^	95.43 (3)	N3—N4—C1	105.93 (7)
O1—Mn1—O1^i^	180.0	C4—N5—N6	105.63 (7)
O2^i^—Mn1—N2^i^	91.07 (3)	N7—N6—N5	111.07 (7)
O2—Mn1—N2^i^	88.93 (3)	N6—N7—N8	106.55 (7)
O1—Mn1—N2^i^	89.76 (3)	C4—N8—N7	108.63 (7)
O1^i^—Mn1—N2^i^	90.24 (3)	C4—N8—H8	134.5 (11)
O2^i^—Mn1—N2	88.93 (3)	N7—N8—H8	116.8 (11)
O2—Mn1—N2	91.07 (3)	N1—C1—N4	110.73 (7)
O1—Mn1—N2	90.24 (3)	N1—C1—C2	123.47 (7)
O1^i^—Mn1—N2	89.76 (3)	N4—C1—C2	125.79 (7)
N2^i^—Mn1—N2	179.999 (2)	C3—C2—C1	122.84 (8)
Mn1—O1—H1A	116.9 (11)	C3—C2—H2	122.3 (9)
Mn1—O1—H1B	125.3 (12)	C1—C2—H2	114.9 (9)
H1A—O1—H1B	106.3 (16)	C2—C3—C4	124.29 (8)
Mn1—O2—H2B	123.3 (13)	C2—C3—H3	121.3 (9)
Mn1—O2—H2A	114.9 (13)	C4—C3—H3	114.4 (9)
H2B—O2—H2A	109.2 (17)	N5—C4—N8	108.11 (7)
H3A—O3—H3B	105.6 (18)	N5—C4—C3	124.43 (8)
C1—N1—N2	104.43 (7)	N8—C4—C3	127.46 (7)
			
C1—N1—N2—N3	0.04 (10)	N6—N7—N8—C4	−0.01 (10)
C1—N1—N2—Mn1	164.68 (7)	N2—N1—C1—N4	−0.02 (10)
O2^i^—Mn1—N2—N3	61.58 (7)	N2—N1—C1—C2	−178.57 (8)
O2—Mn1—N2—N3	−118.42 (7)	N3—N4—C1—N1	−0.01 (10)
O1—Mn1—N2—N3	−33.84 (7)	N3—N4—C1—C2	178.50 (8)
O1^i^—Mn1—N2—N3	146.16 (7)	N1—C1—C2—C3	178.52 (9)
O2^i^—Mn1—N2—N1	−102.42 (8)	N4—C1—C2—C3	0.19 (14)
O2—Mn1—N2—N1	77.58 (8)	C1—C2—C3—C4	−178.84 (8)
O1—Mn1—N2—N1	162.16 (8)	N6—N5—C4—N8	0.29 (10)
O1^i^—Mn1—N2—N1	−17.84 (8)	N6—N5—C4—C3	−179.93 (8)
N1—N2—N3—N4	−0.05 (11)	N7—N8—C4—N5	−0.18 (10)
Mn1—N2—N3—N4	−167.49 (6)	N7—N8—C4—C3	−179.96 (8)
N2—N3—N4—C1	0.03 (10)	C2—C3—C4—N5	177.57 (9)
C4—N5—N6—N7	−0.30 (10)	C2—C3—C4—N8	−2.68 (15)
N5—N6—N7—N8	0.19 (11)		

Symmetry codes: (i) −*x*+1, −*y*, −*z*+1.

### Hydrogen-bond geometry (Å, °)

**Table:**

*D*—H···*A*	*D*—H	H···*A*	*D*···*A*	*D*—H···*A*
O1—H1B···N1^ii^	0.833 (18)	2.021 (18)	2.8419 (10)	168.8 (17)
O1—H1A···O3^iii^	0.823 (18)	1.940 (18)	2.7599 (11)	174.0 (16)
O2—H2B···O3^iv^	0.790 (18)	1.996 (19)	2.7797 (11)	171.4 (18)
O2—H2A···N6^v^	0.81 (2)	2.04 (2)	2.8472 (10)	173.7 (18)
O3—H3A···N5^vi^	0.82 (2)	2.09 (2)	2.8922 (11)	164.2 (19)
O3—H3B···O1	0.80 (2)	2.30 (2)	3.0693 (12)	160 (2)
N8—H8···N4^vii^	0.926 (18)	1.792 (18)	2.7171 (10)	176.6 (16)

Symmetry codes: (ii) *x*−1, *y*, *z*; (iii) −*x*, −*y*+1, −*z*+1; (iv) −*x*+1, −*y*+1, −*z*+1; (v) *x*−1, *y*, *z*+1; (vi) −*x*+1, −*y*+1, −*z*; (vii) *x*+1, *y*, *z*.
