# Tetra­aqua­bis{5-[2-(1*H*-tetrazol-5-yl)ethenyl]pyrazolato-κ*N* ^2^}manganese(II) dihydrate. Corrigendum

**DOI:** 10.1107/S1600536808035836

**Published:** 2008-11-13

**Authors:** Tuoping Hu

**Affiliations:** a Department of Chemistry, North University of China, Taiyuan, Shanxi 030051, People’s Republic of China

## Abstract

The chemical name in the title of the paper by Hu [*Acta Cryst.* (2008), E**64**, m772] is corrected.

## Abstract 2

Corrigendum to *Acta Cryst.* (2008), E**64**, m772.

In the paper by Hu [*Acta Cryst.* (2008), E**64**, m772], the chemical name in the title is incorrect. The correct title should be ‘Tetra­aqua­bis{5-[2-(1*H*-tetrazol-5-yl)ethenyl]tetrazolato-κ*N* ^2^}manganese(II) dihydrate’.

